# Screening Technologies for Target Identification in Pancreatic Cancer

**DOI:** 10.3390/cancers3010079

**Published:** 2010-12-29

**Authors:** Patrick Michl, Stefanie Ripka, Thomas Gress, Malte Buchholz

**Affiliations:** Department of Gastroenterology and Endocrinology, University Hospital, Philipps-University Marburg, Baldinger Strasse, D-35043 Marburg, Germany; E-Mail: gastro@med.uni-marburg.de (T.G.)

**Keywords:** pancreatic cancer, high-throughput screen, microarray, RNA interference, loss-of-function screen

## Abstract

Pancreatic cancer exhibits an extraordinarily high level of resistance to almost any kind of systemic therapy evaluated in clinical trials so far. Therefore, the identification of novel therapeutic targets is urgently required. High-throughput screens have emerged as an important tool to identify putative targets for diagnosis and therapy in an unbiased manner. More than a decade ago, microarray technology was introduced to identify differentially expressed genes in pancreatic cancer as compared to normal pancreas, chronic pancreatitis and other cancer types located in close proximity to the pancreas. In addition, proteomic screens have facilitated the identification of differentially secreted proteins in body fluids of pancreatic cancer patients, serving as possible biomarkers. Recently, RNA interference-based loss-of-function screens have been used to identify functionally relevant genes, whose knock-down has impact on pancreatic cancer cell viability, thereby representing potential new targets for therapeutic intervention. This review summarizes recent results of transcriptional, proteomic and functional screens in pancreatic cancer and discusses potentials and limitations of the respective technologies as well as their impact on future therapeutic developments.

## 1. Introduction

Pancreatic cancer carries the most dismal prognosis of all solid tumors. It is the fourth leading cause of cancer death in the U.S. with approximately 36,800 deaths attributable to pancreatic cancer in 2010 [1]. Since it is rarely detected in early stages and owing to its resistance to drugs and radiotherapy, pancreatic cancer has a poor prognosis, with a five-year survival rate of less than 5% [2].

During the last decades, the genetic alterations underlying pancreatic cancer have been well characterized: 80–95% of pancreatic ductal adenocarcinomas carry activating mutations in the *KRAS2* gene, and 85–98% have mutations, deletions, or hypermethylation in the *CDKN2 (p16)* gene. Furthermore, 50% of the tumors show inactivating mutations of *p53* and about 55% have homozygous deletions or mutations of *SMAD4* [3]. Some of these mutations can already be found in preinvasive precursor lesions of pancreatic cancer, such as pancreatic intraepithelial neoplasias or PanIN lesions, which can be graded according to their histomorphological appearance and their accumulating genetic alterations in PanIN I–III. The initial alterations occurring in early PanIN lesions (PanIN I) include *KRAS2* gene mutations and telomere shortening. During PanIN progression, p16/CDKN2A gets frequently inactivated [3]. Finally, the transition from preinvasive PanIN III lesions to invasive carcinoma is characterized by inactivation of further tumor suppressors such as TP53 and SMAD4/DPC4.

The identification and characterization of these cancer-related genes have increased our understanding of the genetic basis of pancreatic cancer development, but unfortunately this knowledge has not translated into clinical practice, since survival of patients with this disease has not improved significantly over the past two decades. It appears that a multitude of transcriptional and posttranslational events regulate the expression and function of oncogenic and tumor-suppressive genes. To identify new therapeutically exploitable targets differentially expressed in pancreatic cancer, coordinated screening efforts not only at the DNA level, but also at the RNA and protein level are necessary. In addition, screening of proteins for their functional impact on cardinal hallmarks of cancer such as survival, invasiveness and proliferation, e.g., by loss-of-function screens, are mandatory to identify novel functionally relevant targets in an unbiased manner.

## 2. Microarray Screens for Transcriptional Alterations in Pancreatic Cancer

Owing to the fact that hybridization reactions between complementary nucleic acid strands are relatively uniform and well-predictable in their behavior, transcriptome analyses were the natural choice for the development of the first high-throughput screening approaches in molecular biology. To this day, they represent the most widely available and most commonly employed “-omics” technology.

Expression profiling by cDNA-array technology has been introduced almost two decades ago. Originally developed with nylon arrays comprising a few hundred genes in the 1990s, the technology has rapidly advanced and offers now the possibility to perform genome-wide screens on various technological platforms [4]. DNA microarrays are produced by a variety of different techniques using different materials. The common theme is that gene-specific capture probes are immobilized in defined patterns on solid support surfaces, where they can later pair with complementary sequences from an analytical sample. During the experiment, RNAs from test and reference samples are labeled with specific dyes during reverse transcription, resulting in labeled single-strand cDNA which is subsequently hybridized to the cognate sequences spotted on the array. The signal of the bound cDNA at a defined spot can be detected with specific scanners or microscopes, providing a measure of the relative abundance of the corresponding mRNA in the original sample [5].

A typical application of this type of analysis is the comparison of mRNA expression levels detected in tissue samples from malignant tumors with those detected in non-malignant samples from the same organ in order to identify differentially expressed genes which may be suitable as targets for the development of novel diagnostic or therapeutic approaches. During recent years, literally thousands of studies of this type have been performed with virtually every type of malignancy encountered in humans. In pancreatic cancer research, the first expression profile of pancreatic cancer tissues was published by Gress *et al.* as early as 1996 [6]. Since then, several dozen studies of different scale and scope, from specialized target gene selections to global whole-genome transcriptome analyses, have followed [7,8].

The majority of these studies have used bulk tissue as a source for mRNA extraction, which represents a potential pitfall for the interpretation of the data. Pancreatic tumors are often characterized by extensive stromal depositions referred to as the desmoplastic reaction of pancreatic cancer. The stroma may comprise up to 90% of the tumor volume and consists of fibroblasts, stellate cells, vessels and numerous inflammatory cells [9], thus in many aspects resembling the inflammatory processes observed in chronic pancreatitis. Accordingly, expression profiling analyses often detect significantly fewer genes differentially expressed between pancreatic cancer and chronic pancreatitis than between pancreatic cancer and normal pancreas, likely because of the shared stromal influences in the two diseases (see e.g., [10]). In order to circumvent this problem, several groups have employed microdissection techniques to produce samples highly enriched for tumor or control cells, for subsequent microarray analyses [11-14]. Our own group has successfully applied this strategy to the analysis of normal pancreatic ducts, PanIN lesions of different grades, and invasive ductal adenocarcinoma, thus resulting in a comprehensive view of gene expression changes across different stages of the process of tumor formation and progression in the pancreas [15].

Although DNA microarrays are very powerful tools which have profoundly enriched our understanding of transcriptional changes that are associated with malignant transformation of pancreatic cells, they do have a number of limitations inherent to the technology. Chief among them are the need for a-priory knowledge of the sequences one wants to detect on the microarray as well as the inability to completely exclude unspecific cross hybridization events. The advent of next generation sequencing technologies has opened up the possibility to generate expression profiles by sequencing entire cDNA populations generated from a given sample, with the added value of being able to detect unknown genes, splice variants, or mutations present in the sample. As a result, microarray analyses are increasingly being replaced by sequencing-based experimental strategies. As a prominent example, Jones *et al.* have recently published a study combining SNP analyses, exon sequencing and transcriptome sequencing, which led to the definition of 12 “core signaling pathways” that were altered in 67% to 100% of the pancreatic cancer cases they analyzed [16].

Due to the large number of publications and the overwhelming amount of data that have been generated in these studies, it has become virtually impossible for individual researchers to keep an overview of all available transcriptome data and to integrate and interpret their own data in the context of other groups' results. In order to deal with this problem, Chelala and coworkers have developed a web-based resource to systematically collect, annotate and make accessible all publicly available pancreatic expression datasets [17,18]. This continuously updated database, which can be accessed at http://www.pancreasexpression.org/, allows one to comprehensively search for gene expression results across many different studies in a single query. Datasets can be filtered by any attribute desired, e.g., type of samples used in the study, mode of sample preparation, type of array or hybridization protocol used, *etc.* Alternatively, lists of genes can be specified for which all available information can be retrieved. The different types of queries can be freely combined in order to deliver the desired information, thus making this database an invaluable resource for pancreatic cancer researchers.

## 3. High Throughput Proteomic Analyses in Pancreatic Cancer

In contrast to nucleic acids, which, regardless of the type of gene they encode, constitute a relatively uniform class of macromolecules, proteins display an extremely broad range of physicochemical properties. It is thus considerably more challenging to quantitatively recover, accurately separate and unequivocally identify proteins from complex samples on a global scale. Accordingly, several vastly different techniques have been developed and are being employed to perform this task. Detailed descriptions of the different methods and approaches, which include two-dimensional gel electrophoresis (2DE), mass spectrometry based techniques, and antibody or protein microarrays, are given elsewhere [19-21]. None of these is by itself able to offer complete proteome coverage, and the choice of technique as well as the choice of sample preparation protocols (*i.e.*, fractionation techniques, depletion of high abundance proteins, *etc.*) will greatly influence the profile of proteins detected, such that comparisons of results across different studies are very difficult. Furthermore, most of these techniques do not readily lend themselves to high throughput application; thus, the numbers of samples that are analyzed in proteomic studies are often considerably smaller than what is seen in transcriptomic studies.

Nonetheless, taking a direct view at the proteins that are expressed in a given cell type or tissue, as opposed to using mRNA abundance as a rather indirect measure of gene activity, is very attractive in order to elucidate functional differences between cancer and control tissues. Moreover, proteins are potentially very useful biomarkers, since they tend to be much more stable than mRNA in body fluids such as blood, urine, pancreatic or duodenal juice, and, once identified and characterized, can often very sensitively and specifically be detected by antibody-based techniques. Accordingly, many proteome studies performed in pancreatic research have aimed at identifying new protein markers of the disease:

In the analysis of tissue samples to directly assess protein expression in pancreatic cancer, 2DE has historically been the most widely used technique. Tian *et al.* [22] and Qi *et al.* [23] used eight pairs of matched cancer and normal tissue samples, respectively, to identify spots of differentially expressed proteins. Thirty and 48 proteins, respectively, were subsequently identified by mass spectrometry. Only three proteins were commonly detected in both studies, illustrating the variability of results even if similar methodological approaches are taken. In a very recent study, Sitek *et al.* [24] used 2DE followed by mass spectrometry to analyze microdissected PanIN lesions of different grades. Thirty-one non-redundant proteins were identified that significantly change in their expression levels during PanIN progression towards invasive PDAC. Among these candidates, major vault protein (MVP), AGR2, 14-3-3 sigma, ANXA4 and S100A4 were later successfully validated in PanIN lesions by immunohistochemistry. Taking a fundamentally different approach, Chen *et al.* [25] analyzed two pairs of matched normal and pancreatic cancer samples by isotope-coded affinity tag technology and tandem mass spectrometry. One hundred and fifty-one differentially expressed proteins were identified, although the significance of the results is of course limited by the very small number of samples analyzed. Crnogorac-Jurcevic *et al.* [26] used commercially available Nylon filter based arrays of 900 primary antibodies to query pooled samples of pancreatic cancer, chronic pancreatitis and normal pancreas for differentially expressed proteins. A total of 30 and 102 proteins were found to be differentially regulated between chronic pancreatitis and normal pancreas or pancreatic cancer and normal pancreas, respectively. Interestingly, a considerable overlap was observed between both lists, again pointing to similarities in the inflammatory components of both diseases.

As indicated above, a considerable number of studies have examined protein content in different body fluids in an attempt to identify novel biomarkers for non-invasive diagnostic applications. Since blood is easy to obtain and a classic source of protein biomarkers in many diseases, it is not surprising that most of these studies have been aimed at identifying differentially expressed proteins in whole blood, serum or plasma of pancreatic cancer patients and controls, using essentially all proteomic techniques available today [27-35]. Pancreatic or duodenal juice as potentially more specific, but also less readily available diagnostic material has been examined in a number of additional studies [36-39], and most recently, two studies have explored the feasibility of using urine as a substrate for protein-based diagnostic analysis [40,41]. Although a considerable number of promising candidates have been described in these studies, no new marker or panel of markers has actually entered clinical application as of yet. The reason for this is that due to the technical requirements, these studies are often very limited in sample size, so that the results demand careful validation in larger studies. In addition, especially for blood and urine, potential confounding effects stemming from other diseases throughout the body must be carefully examined and excluded before these markers can be considered useful for the clinic. This has recently been very clearly demonstrated by Yan *et al.*, who showed that distinction between pancreatic cancer and benign controls (chronic pancreatitis and healthy subjects) using a panel of plasma protein markers was possible with high precision. However, accuracy dramatically deteriorated when patients suffering from biliary duct obstruction were added to the benign control cohort [42].

Very recently, a novel approach to pancreatic cancer biomarker discovery has been developed to enrich and reliably detect differentially expressed and secreted proteins from cell cultures, which employs stable isotope labeling with amino acids in cell cultures (SILAC) coupled with extensive multidimensional separation coupled with tandem mass spectrometry (MS/MS), which successfully identified several promising targets in pancreatic cancer cells [43-45].

## 4. RNA Interference Based Loss-of-Function Screens

In order to decipher the function of cancer-relevant genes in a high-throughput manner, functional genomic screens have been successfully applied in various cellular systems. During recent years, screens which are performed as so-called forward genetic screens to discover the genes underlying a defined phenotype, have been revolutionized by the advent of RNA interference technology [46].

RNA interference represents a naturally occurring mechanism for post-transcriptional suppression of gene expression using double-stranded RNA (dsRNA) [47]. During evolution, it developed as an ancient defense mechanism against viral invaders. Since its first description in *C. elegans* by Fire and Mello 1998 [48], and its identification in eukaryotes by Elbashir and Tuschl 2001 [49], it evolved as a powerful tool both for the functional characterization of individual genes and for high-throughput screens. Double-stranded RNA molecules in the cell are cleaved by the enzyme complex Dicer resulting in small interfering (si) double-stranded RNA molecules. Subsequently, these siRNAs are recognized by another enzyme complex, the RNA-induced silencing complex (RISC), which targets complementary mRNA molecules for degradation [47]. Artificial introduction of siRNA duplexes into cells can thus silence the expression of selected genes.

**Figure 1.**
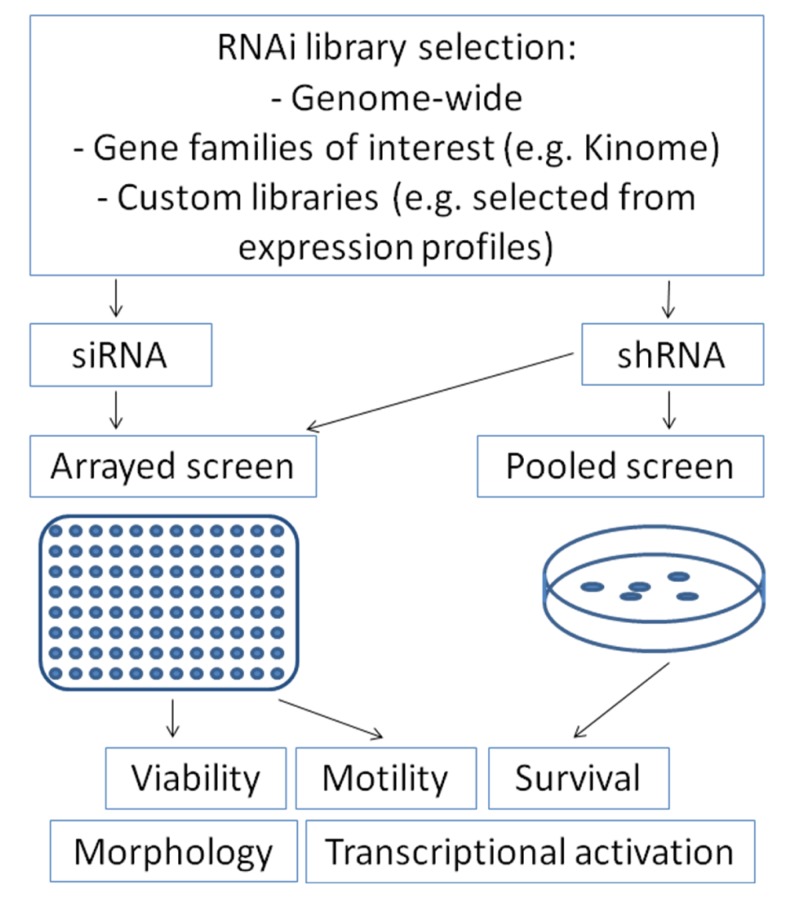
Overview on the use of RNAi libraries in different screening approaches for tumor-relevant read-outs.

RNA interference can be introduced into mammalian cells either by transfection of double-stranded small-interfering RNAs (siRNA) or by transfection or transduction of short-hairpin RNA containing plasmids (shRNA) which are transcribed and subsequently cleaved intracellularly into effective siRNA oligonucleotides by the enzyme Dicer [47]. For screening approaches, RNA interference collections for parallel knock-down of multiple genes are utilized, which may range from gene families of interest (e.g., “Kinome library”) up to genome-wide libraries [50]. These collections contain libraries of siRNA oligonucleotides or shRNA plasmids. Both variants offer distinct advantages and disadvantages, which have to be considered when considering a screen set-up: siRNA libraries allow only transient suppression of the gene of interest, whereas shRNA plasmids containing selection markers can be transfected or transduced stably to allow permanent gene suppression, making long-term assays feasible. In contrast, knock-down efficiency of the gene of interest is frequently more pronounced after siRNA transfection compared to shRNA [51]. Screens may be performed as arrays in 96- or 384-well plate formats with one gene being silenced per well. After introduction of the siRNAs, these plates can be evaluated for many cellular features such as viability, apoptosis, motility, transcriptional activation of distinct pathways using luciferase-based read-outs or cell morphology using high-throughput microscopy (Figure 1). Alternatively, screens may also be performed by transfecting or transducing pooled shRNA-plasmids with selection markers. After selecting transfected cells, enrichment or depletion of distinct shRNAs can be analyzed by high-throughput sequencing.

Several RNA interference-based screens have been performed to identify novel diagnostic or therapeutic targets in pancreatic cancer [52,53]. Giroux *et al.* screened the human kinome for kinases whose inhibition could increase spontaneous or gemcitabine-induced apoptosis in MiaPaCa-2 pancreatic cancer cells. By screening siRNAs directed against 645 kinases, they identified a panel of kinases acting synthetically lethal with gemcitabine including AK1, GRAF, MAP3K7, CSNK2A1, and PAK7, whose inhibition led to significantly enhanced drug-induced apoptosis in several pancreatic cancer cell lines [52].

A second synthetic lethal screen was published recently by Azorsa *et al.* [53]: They utilized a RNAi screen targeting 572 known kinases to identify genes that when silenced would sensitize pancreatic cancer cells to gemcitabine. The greatest potentiation was shown by siRNA targeting checkpoint kinase 1 (CHK1), which could also be targeted with specific small molecule inhibitors thereby significantly enhancing gemcitabine action [53].

Apart from classical synthetic lethal approaches, several combinatorial approaches utilizing RNAi libraries have been published. Microarray-based, genome-wide analysis for DNA copy number aberrations in pancreatic cancer identified several recurrent amplifications, including a 1.1 MB amplicon at 19q13. Based on these data, Kuuselo *et al.* applied a high-throughput loss-of-function screen by RNA interference across this amplicon to identify functionally relevant genes whose down-regulation affected cell viability.

This screen revealed five genes whose knock-down affected cell viability selectively in amplified but not in non-amplified pancreatic cancer cells. Of these, the transcriptional regulator intersex-like (IXL) was consistently overexpressed in amplified cells and had the most dramatic effect on cell viability. IXL silencing also resulted in G0-G1 cell cycle arrest and increased apoptosis, suggesting that IXL is required for cancer cell survival in 19q13-amplified tumors [54,55].

Apart from cell viability, other features of tumor progression can be used as read-out for the screens, such as cell migration, apoptosis, cell morphology or activation of distinct signaling pathways of interest in luciferase-based approaches. Our group identified the transcription factor CUX1/CUTL1 in an shRNA library based screen for mediators of cell motility comprising several hundred genes [56]. We could verify an important role of CUX1 as regulator of cell migration, invasion and survival, associated with high expression levels of CUX1 in several independent cohorts of pancreatic cancer tissues [56,57]. In order to identify functionally relevant downstream targets of CUX1, we applied a sequential approach combining expression profiling and functional genomics using siRNA library. Following cDNA microarray expression profiles, we designed a custom RNAi library targeting 41 putative CUX1 target genes with three silencing sequences each. Using this approach, we identified several CUX1 targets mediating its effects on cell motility and survival, among them the glutamate receptor GRIA3, which has not been shown to affect tumor progression before [58].

These reports utilizing RNAi libraries in different experimental settings demonstrate that RNAi-based loss-of-function screens have evolved as important tools to identify novel potentially relevant targets in pancreatic cancer.

## 5. Outlook

During the last decade, the advent of several new technologies for high-throughput screening has revolutionized research aiming to identify novel targets with potential diagnostic, prognostic, predictive or therapeutic impact. Ranging from RNA expression profiling, which has evolved as a tool with potential impact for therapeutic decision making and response prediction, up to functional screens aiming to decipher the functional consequences of large scale inhibition of gene expression, high- throughput screening offers a unique opportunity to identify novel targets in an unbiased manner. Based on the unprecedented wealth of data which may result from single screening experiments, bioinformatic analysis of the experimental data and target selection remains challenging, which requires careful statistical considerations while setting-up the screen conditions. Hopefully, the novel screening technologies will indeed allow the identification of genes relevant for tumor progression which can be therapeutically targeted, in order to improve the dismal prognosis of pancreatic cancer. However, it has to be emphasized that up to now, candidates identified by these screening approaches have not entered clinical application and therapeutic management has largely been unchanged over the last decades. One reason for this disappointing fact might be the lack of preclinical validation platforms for published screening targets, e.g., by the use of genetically engineered mouse models of pancreatic cancer which could be used to bridge the gap between basic science and clinical advances and drive translational approaches forward.
